# 2-Amino­anilinium 2-chloro­acetate

**DOI:** 10.1107/S1600536810024554

**Published:** 2010-07-07

**Authors:** A. Srinivasa Rao, Bharat Kumar Tripuramallu, Kishore Ravada, Samar K. Das

**Affiliations:** a School of Chemistry, University of Hyderabad, Hyderabad 500 046, India

## Abstract

In the crystal structure of the title compound, C_6_H_9_N_2_^+^·ClCH_2_COO^−^, prepared by the reaction of OPDA (orthophenelynediamine) with chloro­acetic ­acid, N—H⋯O hydrogen bonds generate ladder-like chains and very weak inter­molecular C—H⋯Cl hydrogen-bonding inter­actions between the anions and cations lead to a supra­molecular network. C—H⋯O inter­actions also occur.

## Related literature

For hydrogen bonding with chlorine, see: Brammer *et al.* (2008 ▶); Metrangolo *et al.* (2006 ▶, 2009 ▶). For ladder-like networks, see: Kinbara, Hashimoto *et al.* (1996 ▶); Kinbara, Kai *et al.* (1996 ▶).
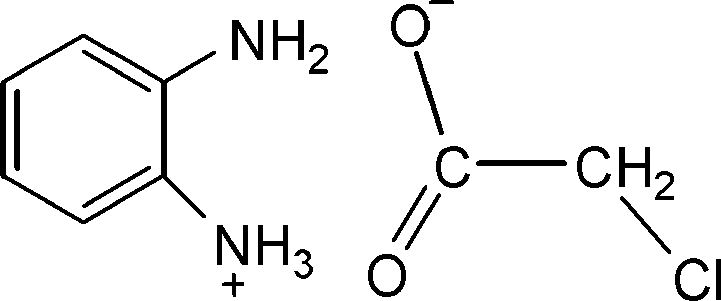


## Experimental

### 

#### Crystal data

C_6_H_9_N_2_^+^·C_2_H_2_ClO_2_^−^
*M* *_r_* = 202.64
Monoclinic, 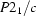

*a* = 11.371 (3) Å
*b* = 4.4852 (11) Å
*c* = 20.115 (4) Å
β = 110.439 (12)°
*V* = 961.3 (4) Å^3^
*Z* = 4
Mo *K*α radiation
μ = 0.37 mm^−1^
*T* = 298 K
0.36 × 0.20 × 0.16 mm

#### Data collection

Bruker SMART CCD area-detector diffractometer
Absorption correction: multi-scan (*SADABS*; Bruker, 2003 ▶) *T*_min_ = 0.879, *T*_max_ = 0.944
9366 measured reflections
1922 independent reflections
1651 reflections with *I* > 2σ(*I*)
*R*_int_ = 0.025

#### Refinement

*R*[*F* ^2^ > 2σ(*F* ^2^)] = 0.047
*wR*(*F* ^2^) = 0.137
*S* = 1.09
1922 reflections
126 parameters
H atoms treated by a mixture of independent and constrained refinement
Δρ_max_ = 0.33 e Å^−3^
Δρ_min_ = −0.29 e Å^−3^

### 

Data collection: *SMART* (Bruker, 2003 ▶); cell refinement: *SAINT* (Bruker, 2003 ▶); data reduction: *SAINT*; program(s) used to solve structure: *SHELXS97* (Sheldrick, 2008 ▶); program(s) used to refine structure: *SHELXL97* (Sheldrick, 2008 ▶); molecular graphics: *SHELXTL* (Sheldrick, 2008 ▶); software used to prepare material for publication: *SHELXTL*.

## Supplementary Material

Crystal structure: contains datablocks I, global. DOI: 10.1107/S1600536810024554/ds2035sup1.cif

Structure factors: contains datablocks I. DOI: 10.1107/S1600536810024554/ds2035Isup2.hkl

Additional supplementary materials:  crystallographic information; 3D view; checkCIF report

## Figures and Tables

**Table 1:** Hydrogen-bond geometry (Å, °)

*D*—H⋯*A*	*D*—H	H⋯*A*	*D*⋯*A*	*D*—H⋯*A*
N1—H1*C*⋯O2^i^	0.90	1.88	2.777 (2)	173
N1—H1*B*⋯O2^ii^	0.94	1.82	2.763 (2)	173
N2—H2*B*⋯O1	0.87	2.16	3.004 (3)	163
C4—H4⋯O1^iii^	0.93	2.66	3.527 (3)	156
C3—H3⋯Cl1^iv^	0.93	3.24	3.985 (3)	138
N2—H2*A*⋯N2^iii^	0.81	2.77	3.587 (4)	179
C8—H8*A*⋯Cl1^v^	0.90 (3)	3.10 (3)	3.878 (3)	146 (3)
C8—H8*B*⋯O1^vi^	0.99 (4)	2.71 (4)	3.491 (4)	136 (3)

Symmetry codes: (i) 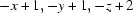; (ii) 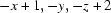; (iii) 
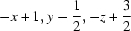
; (iv) 
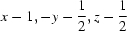
; (v) 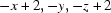; (vi) 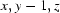.

**Fig. 1.**
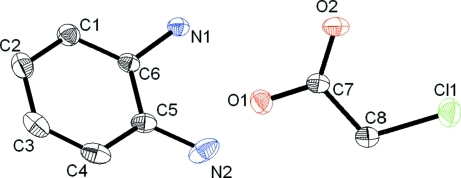
ORTEP diagram of the compound 1 (Thermal ellipsoids are at 50% probability level).

**Fig. 2.**
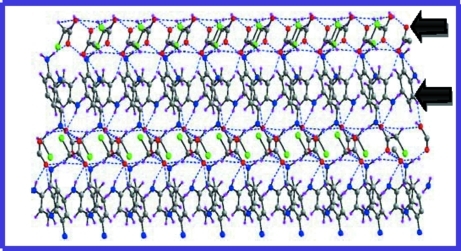
Interactions of C—H···cl in the compound 1 give rise to diverse supramolecular network and all the inetractions arround the cation and anion respectively with symetry codes All the symetry codes for hyderogen bonding were written in the Table 1

**Fig. 3.**
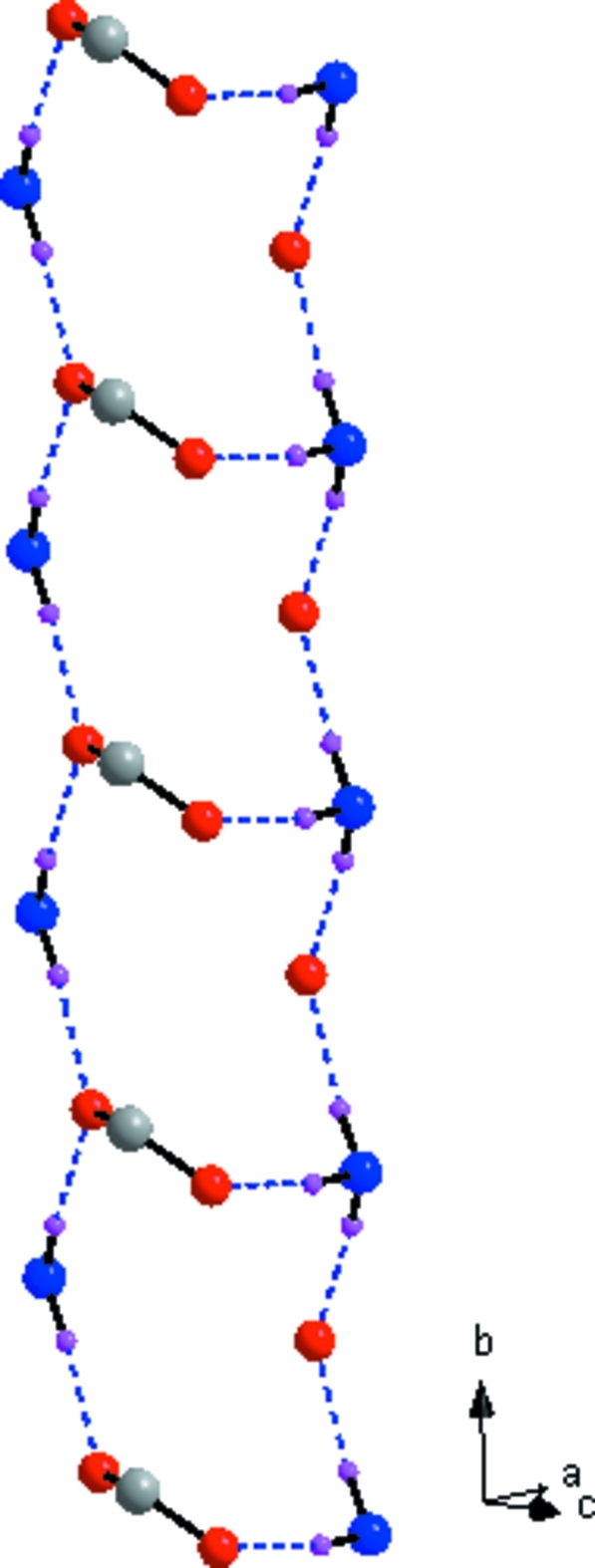
The ladder-type one-dimensional chainlike arrangement generated by N—H···O hydrogen bonding interactions.

**Fig. 4.**
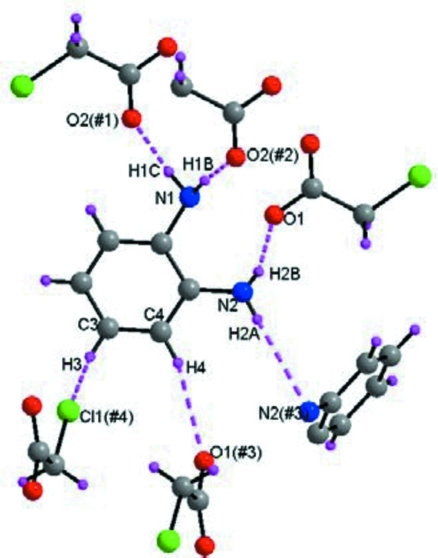
Hydrogen bonding situation around the cation.

**Fig. 5.**
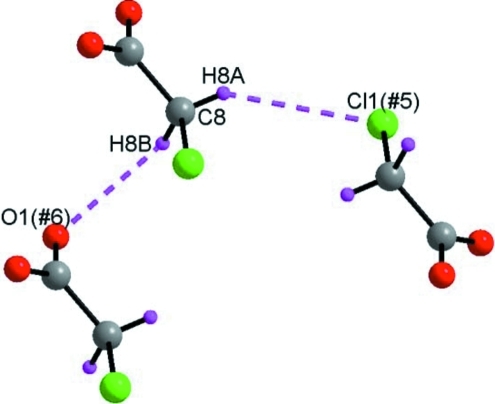
Hydrogen bonding situation around the anion.

**Fig. 6.**
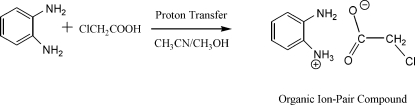
The formation of the title compound.

## supplementary crystallographic information

### Comment

We have reported here the synthesis and structural characterization of a hitherto unknown organic ion pair compound 1, consisting of orthophenylenediammonium cation and chloroacetate anion, that provides a good supramolecular information. The ladder-type one-dimensional chainlike arrangement has been generated because of N—H···O hydrogen bonding interaction in the crystal of compound 1, as shown in Fig. 3.

### Experimental

OPDA (Orthophenelynediamine)(0.108 g, 1 mmol) was dissolved in 20 ml of acetonitrile solution and which was added the solution of 25 ml of methanol containing chloroaceticacid (0.23 g, 1 mmol); this reaction mixture was stirred for 5 min and kept for crystalization at room temperature. Colorless needle-like crystals were formed after 3 days (yield: 0.145 g, 72% based on OPDA).

### Refinement

All H atoms were found on difference maps, with C—H=0.93 Å and included in the final cycles of refinement using a riding model, with *U*_iso_(H)=1.2Ueq(C)

### Crystal data

**Table:**

C_6_H_9_N_2_^+^·C_2_H_2_ClO_2_^−^	*F*(000) = 424
*M**_r_* = 202.64	*D*_x_ = 1.400 Mg m^−^^3^
Monoclinic, *P*2_1_/*c*	Mo *K*α radiation, λ = 0.71073 Å
Hall symbol: -P 2ybc	Cell parameters from 5050 reflections
*a* = 11.371 (3) Å	θ = 2.3–26.1°
*b* = 4.4852 (11) Å	µ = 0.37 mm^−^^1^
*c* = 20.115 (4) Å	*T* = 298 K
β = 110.439 (12)°	Needle, colorless
*V* = 961.3 (4) Å^3^	0.36 × 0.20 × 0.16 mm
*Z* = 4	

### Data collection

**Table:**

Bruker SMART CCD area-detector diffractometer	1922 independent reflections
Radiation source: fine-focus sealed tube	1651 reflections with *I* > 2σ(*I*)
graphite	*R*_int_ = 0.025
phi and ω scans	θ_max_ = 26.2°, θ_min_ = 1.9°
Absorption correction: multi-scan (*SADABS*; Bruker, 2003)	*h* = −14→14
*T*_min_ = 0.879, *T*_max_ = 0.944	*k* = −5→5
9366 measured reflections	*l* = −24→24

### Refinement

**Table:**

Refinement on *F*^2^	Primary atom site location: structure-invariant direct methods
Least-squares matrix: full	Secondary atom site location: difference Fourier map
*R*[*F*^2^ > 2σ(*F*^2^)] = 0.047	Hydrogen site location: inferred from neighbouring sites
*wR*(*F*^2^) = 0.137	H atoms treated by a mixture of independent and constrained refinement
*S* = 1.09	*w* = 1/[σ^2^(*F*_o_^2^) + (0.0676*P*)^2^ + 0.3616*P*] where *P* = (*F*_o_^2^ + 2*F*_c_^2^)/3
1922 reflections	(Δ/σ)_max_ = 0.001
126 parameters	Δρ_max_ = 0.33 e Å^−^^3^
0 restraints	Δρ_min_ = −0.29 e Å^−^^3^

### Special details

**Table:**

Geometry. All e.s.d.'s (except the e.s.d. in the dihedral angle between two l.s. planes) are estimated using the full covariance matrix. The cell e.s.d.'s are taken into account individually in the estimation of e.s.d.'s in distances, angles and torsion angles; correlations between e.s.d.'s in cell parameters are only used when they are defined by crystal symmetry. An approximate (isotropic) treatment of cell e.s.d.'s is used for estimating e.s.d.'s involving l.s. planes.
Refinement. Refinement of *F*^2^ against ALL reflections. The weighted *R*-factor *wR* and goodness of fit *S* are based on *F*^2^, conventional *R*-factors *R* are based on *F*, with *F* set to zero for negative *F*^2^. The threshold expression of *F*^2^ > σ(*F*^2^) is used only for calculating *R*-factors(gt) *etc*. and is not relevant to the choice of reflections for refinement. *R*-factors based on *F*^2^ are statistically about twice as large as those based on *F*, and *R*- factors based on ALL data will be even larger.

### Fractional atomic coordinates and isotropic or equivalent isotropic displacement parameters (Å^2^)

**Table:**

	*x*	*y*	*z*	*U*_iso_*/*U*_eq_	
N1	0.38338 (15)	0.3513 (3)	0.89834 (8)	0.0425 (4)	
H1A	0.4696	0.3585	0.9056	0.051*	
H1B	0.3687	0.1914	0.9249	0.051*	
H1C	0.3625	0.5114	0.9189	0.051*	
N2	0.4570 (2)	−0.0303 (6)	0.80725 (13)	0.0819 (7)	
H2A	0.4758	−0.1428	0.7809	0.098*	
H2B	0.5201	0.0394	0.8428	0.098*	
C1	0.1923 (2)	0.4640 (5)	0.79778 (11)	0.0539 (5)	
H1	0.1695	0.5954	0.8269	0.065*	
C2	0.1128 (2)	0.4187 (6)	0.72902 (12)	0.0641 (6)	
H2	0.0360	0.5167	0.7117	0.077*	
C3	0.1485 (3)	0.2267 (6)	0.68631 (12)	0.0655 (7)	
H3	0.0950	0.1932	0.6400	0.079*	
C4	0.2628 (2)	0.0833 (6)	0.71141 (12)	0.0630 (6)	
H4	0.2861	−0.0422	0.6813	0.076*	
C5	0.3443 (2)	0.1229 (5)	0.78112 (11)	0.0499 (5)	
C6	0.30556 (18)	0.3157 (4)	0.82373 (10)	0.0422 (4)	
Cl1	0.88856 (6)	−0.20415 (18)	1.05309 (4)	0.0787 (3)	
O1	0.63853 (15)	0.3221 (4)	0.92605 (9)	0.0630 (5)	
O2	0.65904 (16)	0.1467 (3)	1.03239 (8)	0.0563 (4)	
C7	0.69161 (19)	0.1690 (4)	0.97966 (10)	0.0453 (5)	
C8	0.8017 (2)	−0.0074 (7)	0.97533 (13)	0.0612 (6)	
H8A	0.855 (3)	0.122 (8)	0.9661 (16)	0.090 (10)*	
H8B	0.767 (3)	−0.161 (9)	0.938 (2)	0.118 (13)*	

### Atomic displacement parameters (Å^2^)

**Table:**

	*U*^11^	*U*^22^	*U*^33^	*U*^12^	*U*^13^	*U*^23^
N1	0.0520 (9)	0.0381 (8)	0.0424 (8)	−0.0007 (7)	0.0229 (7)	−0.0024 (6)
N2	0.0660 (13)	0.0873 (16)	0.0926 (16)	0.0075 (12)	0.0278 (12)	−0.0425 (13)
C1	0.0615 (13)	0.0508 (12)	0.0534 (12)	0.0014 (10)	0.0250 (10)	0.0040 (9)
C2	0.0615 (13)	0.0702 (15)	0.0563 (13)	0.0000 (12)	0.0150 (11)	0.0151 (12)
C3	0.0715 (15)	0.0782 (16)	0.0434 (12)	−0.0223 (13)	0.0160 (11)	0.0026 (11)
C4	0.0819 (17)	0.0650 (14)	0.0515 (12)	−0.0217 (13)	0.0351 (12)	−0.0159 (11)
C5	0.0571 (12)	0.0476 (11)	0.0528 (11)	−0.0117 (9)	0.0290 (10)	−0.0088 (9)
C6	0.0515 (11)	0.0379 (9)	0.0421 (10)	−0.0073 (8)	0.0228 (8)	0.0004 (7)
Cl1	0.0550 (4)	0.0965 (6)	0.0777 (5)	0.0095 (3)	0.0146 (3)	0.0200 (4)
O1	0.0545 (9)	0.0749 (11)	0.0602 (10)	0.0028 (8)	0.0207 (8)	0.0171 (8)
O2	0.0803 (10)	0.0421 (8)	0.0640 (9)	−0.0001 (7)	0.0472 (8)	−0.0018 (6)
C7	0.0480 (11)	0.0445 (10)	0.0471 (11)	−0.0094 (8)	0.0214 (9)	−0.0046 (8)
C8	0.0584 (13)	0.0771 (17)	0.0549 (13)	0.0100 (12)	0.0285 (11)	0.0064 (12)

### Geometric parameters (Å, °)

**Table:**

N1—C6	1.461 (2)	C3—C4	1.378 (4)
N1—H1A	0.9402	C3—H3	0.9300
N1—H1B	0.9425	C4—C5	1.396 (3)
N1—H1C	0.9015	C4—H4	0.9300
N2—C5	1.385 (3)	C5—C6	1.393 (3)
N2—H2A	0.8138	Cl1—C8	1.767 (3)
N2—H2B	0.8747	O1—C7	1.243 (3)
C1—C2	1.378 (3)	O2—C7	1.244 (2)
C1—C6	1.380 (3)	C7—C8	1.509 (3)
C1—H1	0.9300	C8—H8A	0.90 (3)
C2—C3	1.374 (4)	C8—H8B	0.99 (4)
C2—H2	0.9300		
			
C6—N1—H1A	112.9	C3—C4—C5	121.3 (2)
C6—N1—H1B	109.6	C3—C4—H4	119.4
H1A—N1—H1B	108.6	C5—C4—H4	119.4
C6—N1—H1C	113.4	N2—C5—C6	121.6 (2)
H1A—N1—H1C	109.2	N2—C5—C4	121.3 (2)
H1B—N1—H1C	102.6	C6—C5—C4	117.1 (2)
C5—N2—H2A	118.7	C1—C6—C5	121.37 (19)
C5—N2—H2B	121.3	C1—C6—N1	119.11 (17)
H2A—N2—H2B	115.2	C5—C6—N1	119.45 (18)
C2—C1—C6	120.5 (2)	O1—C7—O2	125.8 (2)
C2—C1—H1	119.8	O1—C7—C8	113.63 (18)
C6—C1—H1	119.8	O2—C7—C8	120.5 (2)
C3—C2—C1	119.1 (2)	C7—C8—Cl1	115.41 (16)
C3—C2—H2	120.4	C7—C8—H8A	107 (2)
C1—C2—H2	120.4	Cl1—C8—H8A	107 (2)
C2—C3—C4	120.7 (2)	C7—C8—H8B	107 (2)
C2—C3—H3	119.7	Cl1—C8—H8B	106 (2)
C4—C3—H3	119.7	H8A—C8—H8B	114 (3)
			
C6—C1—C2—C3	−0.8 (3)	N2—C5—C6—C1	−178.9 (2)
C1—C2—C3—C4	−0.7 (4)	C4—C5—C6—C1	−0.9 (3)
C2—C3—C4—C5	1.5 (4)	N2—C5—C6—N1	−1.8 (3)
C3—C4—C5—N2	177.4 (2)	C4—C5—C6—N1	176.18 (18)
C3—C4—C5—C6	−0.7 (3)	O1—C7—C8—Cl1	174.88 (19)
C2—C1—C6—C5	1.6 (3)	O2—C7—C8—Cl1	−7.3 (3)
C2—C1—C6—N1	−175.42 (18)		

### Hydrogen-bond geometry (Å, °)

**Table:**

*D*—H···*A*	*D*—H	H···*A*	*D*···*A*	*D*—H···*A*
N1—H1C···O2^i^	0.90	1.88	2.777 (2)	173
N1—H1B···O2^ii^	0.94	1.82	2.763 (2)	173
N2—H2B···O1	0.87	2.16	3.004 (3)	163
C4—H4···O1^iii^	0.93	2.66	3.527 (3)	156
C3—H3···Cl1^iv^	0.93	3.24	3.985 (3)	138
N2—H2A···N2^iii^	0.81	2.77	3.587 (4)	179
C8—H8A···Cl1^v^	0.90 (3)	3.10 (3)	3.878 (3)	146 (3)
C8—H8B···O1^vi^	0.99 (4)	2.71 (4)	3.491 (4)	136 (3)

Symmetry codes: (i) −*x*+1, −*y*+1, −*z*+2; (ii) −*x*+1, −*y*, −*z*+2; (iii) −*x*+1, *y*−1/2, −*z*+3/2; (iv) *x*−1, −*y*−1/2, *z*−1/2; (v) −*x*+2, −*y*, −*z*+2; (vi) *x*, *y*−1, *z*.
